# Impact of an electronic medium delivery of warfarin education in a low income, minority outpatient population: a pilot intervention study

**DOI:** 10.1186/s12889-019-7370-4

**Published:** 2019-08-05

**Authors:** Krista Heinrich, Katherine Sanchez, Cecilia Hui, Kiara Talabi, Marlena Perry, Huanying Qin, Hoa Nguyen, Amulya Tatachar

**Affiliations:** 1 Department of Clinical Pharmacy, Health Texas Provider Network, Baylor Scott & White Health, 2001 Bryan Street, Suite 2800, Dallas, TX 75201 USA; 2 Center for Applied Health Research, Baylor Scott and White Research Institute, 8080 North Central Expressway, Suite 1050, Dallas, TX 75206 USA; 3 Department of Quantitative Sciences, Baylor Scott and White Health, 8080 N. Central Expressway, Suite 900, Dallas, TX 75206 USA; 4 University of North Texas System College of Pharmacy, University of North Texas Health Science Center, 3500 Camp Bowie Blvd, Fort Worth, TX 76107 USA

**Keywords:** Warfarin, Education, Technology, Primary care, Health literacy

## Abstract

**Background:**

Warfarin is classified as a high-alert medication for ambulatory healthcare and safe guards for high-alert medications are necessary, including the practice of mandatory patient education. The high cost of hospitalizations related to adverse events combined with the average bleeding event rate of 7–8% in spite of routine patient education, suggests the importance of new approaches to standardized health education on warfarin. We sought to evaluate the impact of a warfarin educational video using an electronic tablet on patient knowledge and to determine patients’ satisfaction with the use of an electronic tablet for educational purposes in outpatient clinics serving a low income, minority population.

**Methods:**

A warfarin educational video delivered on an electronic tablet (iPad) was delivered at two pharmacist-managed anticoagulation clinics to uninsured patients whose annual income is equal or less than two hundred percent below the poverty level were offered. Patients (*n* = 18) completed a pre-video and post-video knowledge test on warfarin before and after viewing the warfarin educational video on an electronic tablet and a follow-up test to measure the retention of knowledge and a patient satisfaction survey at 60 days. The primary outcome was change in knowledge test scores. Other outcome measures included adherence rates, adverse events, time in therapeutic INR range, and patient-reported satisfaction scores.

**Results:**

The majority of patients were uninsured men taking warfarin for atrial fibrillation (*n* = 5). The median scores at post-video knowledge test and follow-up knowledge test were significantly higher than that for the pre-knowledge test (12 (11–12) vs. 10(8–11), *p* < 0.001). The study group had a ‘time in therapeutic INR’ range of 56.3%, a rate of adverse events of 24.5%, and a self-reported adherence rate to warfarin of 94.1%. The majority of patients also had positive responses to the patient satisfaction survey.

**Conclusions:**

Patient education delivered via iPad to facilitate knowledge of medication can serve as a useful tool for educating patients about warfarin and warfarin therapy. Use of an electronic medium may be a unique way to provide standard medication education to patients.

**Trial registration:**

The study was retrospectively registered with: NCT03650777; 9/18/18.

## Background

### Warfarin safety and patient education

According to the Institute for Safe Medication Practices (ISMP), warfarin is classified as a high-alert medication for ambulatory healthcare [1]. This is mainly due to its narrow therapeutic index, which requires close follow up in clinics to test the International Normalized Ratio (INR), used to determine if the warfarin dose is within therapeutic range. Despite the development of novel oral anticoagulants, warfarin’s affordability makes it a preferred option for many clinics serving the uninsured, low income patient population. Although its effectiveness as an anticoagulant has been proven for several decades, there is still potential for serious injury and added costs, when warfarin is not appropriately used [1, 2]. A study conducted in 2010 of 126 warfarin-related bleeding hospitalizations found the mean cost to be $10, 819 [3].

The ISMP encourages safe guards for high-alert medications, including the practice of mandatory patient education [1]. Traditional educational materials for warfarin education are verbal counseling and printed pamphlets. Two validated assessments, the Oral Anticoagulant Knowledge (OAK) test and the Anticoagulation Knowledge Assessment (AKA), both written at a 7th grade reading level, currently exist to evaluate patient knowledge of oral anticoagulants [4, 5]. Shrestha et al. reported 94.1% of enrolled patients on warfarin therapy receiving traditional counseling methods failed to achieve a passing score using the AKA questionnaire [6].

The high cost of hospitalizations related to warfarin adverse events combined with the average bleeding event rate of 7–8% in spite of routine patient education, suggests the importance of new approaches to standardized health education on warfarin [3]. Though limited evidence exists on standardization and delivery of warfarin education [7], there is a need for innovative, comprehensive, and timely education to improve patient knowledge on warfarin therapy.

### Electronic media and health education

The Agency for Healthcare Research and Quality (AHRQ) Health Information Technology (Health IT) initiative promotes integration of information technology in health care [8]. Electronic media, such as the iPad, may provide a unique educational opportunity that integrates modern technology into healthcare settings in order to increase patient knowledge of their disease and its treatment. However, only a few studies have investigated the use of electronic media, such as an iPad, to facilitate warfarin education [9–11]. Kim, et al. [9] documented the use of an iPad to distribute warfarin education in an inpatient setting and found that patients increased their knowledge on warfarin therapy upon viewing the video on the iPad.

While there is limited data on the use of electronic media in the inpatient setting, to our knowledge, no studies have tested iPad technology to facilitate warfarin education in outpatient facilities. The authors investigated a unique opportunity to test the impact of a warfarin educational video delivered through an electronic tablet specifically targeting an uninsured, low literacy patient population.

### Health education and literacy

Health literacy refers to an individual’s capacity to understand health related information [12]. The role of patient education in management of chronic disease to increase patient engagement and improve satisfaction and optimize health outcomes has been well established [13]. Low health literacy is related to poor management of chronic diseases, lack of basic knowledge about medical conditions and treatments, less understanding and use of preventive services, worse health outcomes, and higher rates of hospitalization and emergency care use [14].

Patient education programs which address social determinants of health improve a populations’ access to information and their capacity to use it effectively, ultimately increasing patient decision-making and empowerment [15]. Provision of patient education materials that are linguistically, idiomatically, and literacy-level appropriate is key [16, 17]. In the current study, we sought to evaluate the impact of a warfarin educational video delivered using an electronic tablet (iPad) on patient knowledge and satisfaction in a network of outpatient primary care clinics that target uninsured, underserved patients from the community and hospitals following discharge. Specifically, we sought to examine changes in knowledge, rates of self-reported adherence, adverse events, time in therapeutic INR range, and patient-reported satisfaction with the use of an electronic tablet to learn about warfarin.

## Methods

### Setting and participants

The Baylor Scott & White Community Care Clinics consist of 9 interdisciplinary outpatient primary care clinics serving approximately 9000 uninsured patients annually in the Dallas-Fort Worth metroplex. The clinics consist of providers, medical assistants, nurses, community health workers, social workers, community navigators, and a medication management team. The medication management team includes a clinical pharmacy manager, two full-time equivalent (FTE) clinical pharmacists, one part-time clinical pharmacist, two FTE pharmacy technicians, and two Postgraduate Year 1 (PGY1) Community Pharmacy residents.

In February 2016, in response to inconsistent education and poor patient assessment by the clinic staff, Baylor Scott & White pharmacists and pharmacy residents initiated two anticoagulation clinics. Between February 2016 and June 2016, adult (age 18 or older), English-speaking clinic patients currently on warfarin therapy and receiving PT/INR follow-up visits were invited to participate in the study. Patients were excluded if they declined counseling through the use of an iPad, if English was not their first language, or if the patient had a cognitive dysfunction or impairment that prevented full comprehension of the warfarin education. A total of 18 patients consented to receive warfarin education via the iPad. None of these patients were naïve to warfarin and were all previously managed by the medical assistants and/or nurses.

Anticoagulation services at two anticoagulation clinics occurred once a week, were staffed either by a pharmacy resident or pharmacist and served approximately 50 adult (age 18 or older) patients on warfarin therapy during the study period. In addition to making dose adjustments and monitoring patients on warfarin therapy, the pharmacists and residents spent considerable time educating patients on warfarin therapy. The average time spent by a pharmacist for an anticoagulation visit was 15 to 20 min. If the patient was naïve to warfarin, or had not yet been educated by a pharmacist, the visit was 30 min.

A PGY1 community pharmacy resident developed an 18-page warfarin educational booklet to standardize education at the anticoagulation clinics. The content of the educational booklet was based on the American Heart Association “A Patient’s Guide to Taking Warfarin” and the warfarin patient product and full prescribing information [18, 19]. An English and Spanish version of the warfarin educational booklet was reviewed and approved by the quality and compliance department at Baylor Scott & White Health and incorporated into the standard education workflow for the anticoagulation clinics. The Flesch-Kincaid Grade Level was 8.1 [20]. The booklet content included: pathophysiology of thromboembolic disorders, pharmacology of warfarin including mechanism of action and onset of action, indications for therapy, appropriate administration, monitoring (i.e. INR) and dosing, adverse events, signs and symptoms of serious bleeding and clotting events, lifestyle recommendations, and drug- and food-interactions [18]. The average time to educate a patient using a booklet was 15 min.

### Intervention

A warfarin educational video was developed by a PGY1 community pharmacy resident and pharmacy preceptors. The video was hosted on social media (YouTube). Both anticoagulation clinics utilized an iPad to show the warfarin educational video to patients. The video included various auditory and visual stimuli (i.e., animated pictures, inanimate objects, and scene changes). The content and format of the video closely followed the warfarin educational booklet. The video was reviewed and approved through institutional legal and compliance staff, two clinical pharmacists trained in anticoagulation, and the clinical pharmacy manager. The video was edited and finalized by the corporate marketing department at Baylor Scott & White Health. The final video had an approximate run time of 10 min.

The video consisted of an English-speaking pharmacy resident elaborating on six subjects discussed in the warfarin educational booklet: diet, procedures, drug interactions, adverse events, INRs, and administration. The video started with a brief introduction, disclaimer, and explanation of purpose. The video transitioned into a discussion of warfarin pharmacology and role in therapy using animated pictures, including mechanism of action and onset of action, major indications for warfarin, and contraindications (i.e., pregnancy). The video then discussed appropriate administration including management of missed doses and strategies for medication adherence. The next segment included information about warfarin tablet strengths and their respective tablet colors. The video reviewed the refill process to prevent missed doses and promote adherence to warfarin therapy. The next segment focused on monitoring, including the role of INR, INR goal ranges, and the association between INR and bleeding and clotting events. The video then discussed common side effects of warfarin and signs and symptoms of bleeding and clotting. The video also stated emergency room precautions and warnings.

The video focused special attention on various interactions with warfarin including medications, diet, and lifestyle. The video also contained tips for patients to stay safe while on warfarin, such as using gardening gloves and wearing sturdy shoes. There were also instructions to notify all providers, including dentists, of their warfarin therapy and to notify the clinic staff of any upcoming procedures or surgeries. The video concluded with a brief summary of key points about warfarin therapy.

#### Procedures

The Baylor University Medical Center Institutional Review Board approved the study as a Quality Improvement Project. Consented patients independently completed a pre-video knowledge test and then viewed the warfarin educational video on the iPad in a patient care room. The pharmacist was not present during the viewing of the video but was available for any technical difficulties. The pharmacist completed the anticoagulation visit and adjusted warfarin therapy as appropriate. At the conclusion of the visit, the patients independently completed the post-video knowledge test. The pharmacist would then review the knowledge test to clarify any questions or doubts about warfarin therapy and, lastly, provide patients with the warfarin educational booklet. Pharmacists relied heavily on the video to provide thorough education to study participants and did not provide extensive verbal counseling.

Patients viewed the educational video once, at the time of study enrollment. The video and pre- and post-video knowledge tests added an additional 30–40 min to the anticoagulation visit. Patients completed the knowledge test and a satisfaction survey at a follow-up visit within 60-days of study enrollment, in between these visits patients did not have access to the video training again.

### Measures

Data collection included patient demographics, comorbidities, concomitant medications, indication for warfarin therapy, time in therapeutic INR range, warfarin dose adjustments (if applicable), adherence, adverse events, diet, results from the patient satisfaction survey, and scores from the initial visit and follow-up visit knowledge tests.

#### Warfarin therapy knowledge

The knowledge test was adapted from the validated Anticoagulation Knowledge Assessment (AKA) [4] and was administered pre-video, post-video, and at the follow up visit. Twelve questions of 29 questions from the AKA were chosen by the lead clinical pharmacist to 1) specifically assess patient knowledge of the six key areas highlighted in the educational video, and 2) ensure feasibility due to time constraints of clinic visits. The highest possible score was 12 and lowest possible score was 0. Omitted questions did not pertain to the educational video. The questions were multiple choice and assessed patients’ knowledge on: diet, procedures, drug interactions, adverse events, INR definition, and administration. Missed questions on this assessment indicated areas for which the patient lacked knowledge of their warfarin therapy.

#### Satisfaction survey

The satisfaction survey consisted of four Likert-based questions on a scale of 1 (strongly disagree) to 5 (strongly agree) and one open-ended question inquiring about improvements in the anticoagulation clinic (Table 3). The survey assessed patients’ perceptions of the iPad technology, confidence in their knowledge about warfarin therapy, likelihood of recommending this service to fellow patients, and areas where the anticoagulation service could improve. Completed surveys were given to the pharmacist or resident.

#### Adherence and adverse events

The anticoagulation clinic monitored adherence and adverse events at each visit through patient self-report. Patients who denied missed doses of warfarin throughout the study period were considered “adherent.” Patients who reported at least one missed dose of warfarin throughout the study period were considered “non-adherent.” Adverse events were reported by patient at each visit with pharmacist.

### Data analysis

The main outcome evaluated was the pre-video and post-video knowledge test scores. The secondary outcomes included: follow-up knowledge test scores, rates of self-reported adherence, adverse events, time in therapeutic INR range, and patient-reported satisfaction scores. Outcomes were calculated as percentage of visits with INR value within patient’s goal INR range, adverse event, and adherence for each patient. Time in Therapeutic Range (TTR) was assessed using the Rosendaal method, in which the INR value is assumed to change linearly between INR checks [21]. Descriptive statistics were calculated for patient demographics and clinical variables. Satisfaction surveys and knowledge test scores at three time points (pre- and post-intervention, and follow up) were compared using the non-parametric Skillings- Mack test due to missing data issue at follow up visit (data available for 12 out of 18 patients at follow up visit). Pair comparisons between any two of the three time points were performed with a non-parametric test, Wilcoxon signed-rank test, and all *p* values were adjusted using Bonferroni method for multiple comparisons. SAS Enterprise Guide 6.1 was used for data analysis.

And while the sample size was small, the data was sufficient for a preliminary analysis of knowledge, satisfaction, and change over time in a pragmatic trial.

## Results

### Sample

A number of noteworthy sample characteristics were identified. The intervention sample was primarily male (78%, *n* = 14), and majority were minority in ethnicity (61%, *n* = 11). The most common comorbid illness was hypertension (76.5%, *n* = 13) and the most common indication for warfarin therapy was atrial fibrillation (27.8%, *n* = 5). All baseline demographics and clinical variables can be found in Table 1.

**Table 1:** Demographic and clinical characteristics, *N* = 18

Variable	Participants n (%)
Gender	
Male	14 (77.8)
Ethnicity	
White	7 (38.9)
Hispanic	2 (11.1)
African American	7 (38.9)
Other	2 (11.1)
Number of Medications	M (SD) 6.8
Comorbidities	n (%)
Hypertension	14 (77.8)
Hyperlipidemia	7 (38.9)
Diabetes Mellitus	3 (16.7)
Congestive Heart Failure	6 (33.3)
Depression	4 (22.2)
Coronary Artery Disease	5 (27.8)
Chronic Obstructive Pulmonary	5 (27.8)
Disease	
	n (%)^a^
Indication for Warfarin Therapy	5 (27.8)
Atrial fibrillation	4 (22.2)
Pulmonary embolism	4 (22.2)
Deep vein thrombosis	3 (16.7)
Heart valve	2 (11.1)
Mural thrombus	

^a^One patient had more than one indication

All patients (100%, *n* = 18) completed the pre- and post-video assessments. Summary of the scores for three time points of assessment were presented in Table 2. The median (IQR) scores at post-video knowledge test were significantly higher than that of the pre-knowledge test (12 (11–12) vs. 10(8–11), *p* < 0.001).

**Table 2:** Comparison of Knowledge Test Scores*

Variable	Median (IQR)	*P* value for overall Comparison*	Pair Comparison	*P* value for pair comparison§
Pre-knowledge test	10 (8–11)	< 0.001	Post vs Pre	< 0.001
Post-knowledge test	12(11–12)		FU vs Post	1.00
FU-knowledge test	12(10–12)		FU vs Pre	0.005

*IQR Interquartile range

*p value for overall comparison for knowledge test score among 3 time points using Skillings- Mack test

§Bonferroni adjusted *p* value for pair comparison for knowledge test score between time points using Wilcoxon signed-rank test

Twelve patients (66.67%) completed the follow-up knowledge test. The median (IQR) score for the follow-up knowledge test was greater than the pre-knowledge test (12 (11–12) vs. 10(8–11), *p* = 0.005), but was unchanged from the post-knowledge test (Table 2). The most commonly missed questions were questions 4, 6, and 11 which assessed knowledge on management of missed doses and the impact of beverages, including alcohol, on PT/INR lab values.

Table 3 provides patient satisfaction scores completed by 12 of the 18 patients (66%). The majority of patients noted satisfaction with the iPad and quality of the warfarin educational video. The patients also reported confidence in their knowledge of warfarin therapy and likelihood of recommending the educational video to other patients. Furthermore, we found no differences in the patient satisfaction with the iPad delivery as it relates to race/ethnicity, sex, age, or other characteristics.

**Table 3:** Patient Satisfaction Survey Responses

Variable	Mean (SD)
How much did you like using the iPad video?	4.5 (0.5)
How would you rate the quality of the iPad video?	4.3 (0.6)
How confident are you in your knowledge of your warfarin therapy?	4.5 (0.5)
How likely are you to recommend other patients to use the iPad video?	4.5 (0.5)

The secondary outcomes analyzed included time in therapeutic range, adverse events, and medication adherence are presented in Table 4. The study group had a ‘time in therapeutic INR’ range (TTR) of 56.3%. Of the study participants, only 1 reported an adverse event (ADE), subjects could have reported headache, dizziness, shortness of breath, chest pain, swelling in the extremities, blood in stool, nose bleed, gum bleeding, or bruising. Of the subjects who were able to complete a follow up visit, 12 (92.3%) of the patients reported complete adherence to warfarin therapy (i.e. no missed doses).

**Table 4:** Secondary outcomes

Variable	
INR in range	Mean (SD) 56.3 (22.6)
ADE	n(%) 1 (7.7)
Adherence	12 (92.3)

## Discussion

In this sample of uninsured, low literacy patients, we found the use of an electronic tablet to facilitate medication education led to an increase in patient knowledge of their warfarin therapy. Additionally, we found sustained retention of important clinical information 60 days after viewing the warfarin educational video. To the authors’ knowledge, this is the first project of its kind investigating the use of an iPad to deliver warfarin education in an outpatient clinic setting among uninsured, low literacy patients. Our findings are consistent with a similar study performed in a hospital setting [9] which resulted in an increase in patient knowledge, but novel in the delivery via educational video among a unique, outpatient population.

Our findings related to time in therapeutic INR range (TTR) suggest that education of warfarin via an iPad is effective for maintaining TTR to 50% or greater. This finding is similar to those of Palareti et al. [22] and the TREAT (**TR**ial of an **E**ducational intervention on patients’ knowledge of Atrial fibrillation and anticoagulant therapy, INR control, and outcome of **T**reatment with warfarin) study, which also found greater TTR for patients that underwent an intensive, focused educational program compared to usual care [23]. While our project could not definitively demonstrate improvement in TTR rates, the newly established anticoagulation clinic iPad education did show early promise for improving TTR rates, which have been associated with improved patient outcomes, safety and drug efficacy [24].

Patients receiving warfarin education through a handheld tablet appeared to demonstrate self-reported adherence to warfarin therapy, high satisfaction in receiving warfarin education via iPad, and found the video to be high quality and recommended for other clinic patients. Our results are similar to those of Kim, et al., in which the subjects found that the iPad was easy to use and they liked using it [9]. They also found that women preferred using the iPad more than men, whereas we found no differences between men and women on the likeability of the iPad. In fact, our results seem to indicate a high satisfaction rate among all patients who used the iPad to view the warfarin educational video.

Use of electronic devices to facilitate education offers the advantage of educating patients when healthcare professionals have time constraints, the option to replay and repeat information on the video, and the elimination of human error in the delivery of important information. In terms of technology, the iPad provides ease of portability, compared to a television and DVD player. Certainly, there are disadvantages to exclusively using an iPad for education, including the inability to clarify concepts that are not understood, technology failures including poor internet connectivity, volume constraints, and occasional charging issues in addition to the added expense of iPads and lack of take-home literature for patients.

### Limitations

Our study has some limitations. The pharmacist managed anticoagulation clinic occurred only once a week and in only two out of nine anticoagulation clinics which resulted in a small number of patients and lack of comparator group. We also did not collect the total number or reason for patients who declined our iPad educational video which may have helped to discover additional barriers to this type of education medium. Our study did not enroll patients who were naïve to warfarin, which could have affected the initial and follow-up knowledge tests. Administering two knowledge tests and the warfarin video added considerable time to the anticoagulation visit, and therefore one reason to adapt the AKA was for feasibility purposes. The adapted AKA was not adapted for this study. Exclusion criteria also limited the intervention group size. The clinics serve a large Spanish-speaking population and a warfarin video in Spanish could increase the sample size and external validity of our project. However, due to budget and time restraints a video translated in Spanish was not feasible for this pilot project.

Self-reported adherence rates can introduce recall-bias, but data supports that self-reported adherence has moderate correspondence to other adherence measures [25]. The study investigators also utilized INR levels as another means to assess adherence in addition to efficacy. Future studies can consider other direct methods of adherence assessment such as medication cap alarms and directly observed therapy. Finally, this is a feasibility/pilot study with a small sample size from 2 clinics from a health care system, interpretation of the findings should be with caution. Future studies with large sample sizes from multiple facilities are certainly needed.

## Conclusion

Patient education delivered via iPad to facilitate knowledge of medication can serve as a useful tool for educating patients about warfarin and warfarin therapy. Electronic media represents a unique way to provide standard education to patients; however, further studies are needed to prospectively compare traditional warfarin education (verbal counseling and printed pamphlets) with warfarin education delivered through popular, handheld devices. While access to iPads or tablets may be limited for primary care settings, our study suggests an analysis of the return on investment for the purchase of these electronic media tools may be warranted.

## Data Availability

The datasets used and/or analyzed during the current study are available from the corresponding author on reasonable request.

## Abbreviations

AHRQ: Agency for Healthcare Research and Quality
AKA: Anticoagulation Knowledge Assessment
INR: International Normalized Ratio
ISMP: Institute for Safe Medication Practices
PGY1: Post-Graduate Year One
